# Profiles of women who have suffered occupational accidents in cleaning: perceived health, psychosocial risks, and personality variables

**DOI:** 10.1007/s00420-022-01927-8

**Published:** 2022-10-18

**Authors:** Iván Fernández-Suárez, José J. López-Goñi, Begoña Haro

**Affiliations:** 1 Escuela Superior de Ingeniería Técnica, International-University of La Rioja, Logroño, Spain; 2 Departamento de Ciencias de la Salud, Universidad Pública de Navarra, Campus de Arrosadía s/n, 31006 Pamplona, Spain; 3 IdiSNA, Instituto de Investigación Sanitaria de Navarra, Pamplona, Spain

**Keywords:** Accident rate, Working women, Health perception, Risk behaviour, Psychosocial risk

## Abstract

**Purpose:**

The main goal was to identify the variables (sociodemographic, work, psychosocial, perceived health, and personality) associated with occupational accidents suffered in the past by women in the cleaning sector.

**Methods:**

A sample of 455 women was evaluated.

**Results:**

A total of 23.5% of the workers (*n* = 107) had suffered an occupational accident with medical leave. In general, women who had suffered some accident in their life had a worse situation in all areas evaluated. Two subsamples of women had a greater association with accidents. Specifically, the presence of work accidents was 15.9 times higher among those who presented a worse perception of their physical effort and a greater tendency towards risky behaviours and 13.5 times higher among those who had a moderate perception of physical exertion and a disability.

**Conclusion:**

In general, the characteristics of female workers were found to be associated with different accident rates. Preventive actions should be designed individually.

## Introduction

It is estimated that the economic cost of temporary disability (professional contingency and common contingency) is up to 4% of world gross domestic product (Takala et al. 2014). In Spain, absenteeism due to temporary disability represents 13.09 days lost for each active worker. Every day, 753,000 workers are absent from their jobs, translating into an absenteeism rate of 5.3% of the active population (Blasco de Luna et al. 2019).

Within occupational absenteeism, accident rates are one of the great social, labour, and governmental concerns (Hallowell 2010; Lund and Aarø 2004; Althomali 2022). In Spain, both accidents with medical leave (the worker cannot go to work) and accidents without medical leave (the worker, after medical care can be reinstated) imply significant losses for organizations. In 2017 alone, there were 596,606 work accidents with medical leave, representing an increase of 5.6% over the previous year. These accidents generated an average of 31.1 days lost for each process (Ministerio de Trabajo Migraciones y Seguridad Social, 2018). The service sector accumulated a 3.6% absenteeism rate due to temporary disability in 2017, lower than only the industrial sector (3.7%); this rate represents an increase of 46.2% since 2000, making service the sector with the greatest increase (the national average stands at 35.0%) (Blasco de Luna et al. 2019).

To date, different types of approaches to understand the problem of work-related accidents have been taken. One such approach is the tracking and monitoring of accidents that occur. In this type of research, in addition to assessing the evolution in the number of accidents and the possible impact of the measures taken on such accidents, differences in the accident rate related to individual variables such as age or type of contract are examined. Regarding age, higher rates of accidents have been found at the extremes of the age range (in young people due to greater carelessness and in elderly people due to a possible loss of mental–physical conditions) (Balderrama et al. 2015). With regard to the type of contract, the loss ratio has been associated with precarious work and contractual instability (Benach et al. 2014; Romero Caraballo 2019; Simões et al. 2019).

Other lines of research have analysed work conditions collectively but without assessing personal perceptions (Johannessen et al. 2015). In this type of study, for example, the influence of the work environment on worker behaviour (Lee 2022; Lim et al. 2021) or on worker health conditions has been evaluated. These studies have concluded that workers tend to adopt behaviours similar to those of the people around them; therefore, an adequate preventive culture can improve the individual’s safety behaviour and consequently reduce the level of accidents. With respect to health, in an organization’s practice, the health status of its members is usually addressed from an objective perspective through occupational health screening. The conclusions of these exams are focused merely on the aptitude criterion, the ability (eminently physical) of the person to perform the task (Jaúregui-Apellániz 2014). Furthermore, there are other factors that could play an important role such as psychosocial risks that must be considered to preserve health and safety at workplace and to prevent work-related accidents. Psychosocial risks are the interactions between the content of the position, the work organization and management, and other environmental and organizational conditions, on the one hand, and the competencies and needs of employees on the other (Hultén et al. 2022; Helgesson et al. 2020). In fact, recent investigations support the influence of psychosocial risks on workers' health and its association with disorders, such as burnout, mobbing, stress, or depression (Roohani and Iravani 2020; Rayan et al. 2019).

Other studies have been interested in the influence of personality on work accidents (Af Wåhlberg et al. 2015; Li et al. 2015; Mullan et al. 2015). People under identical work conditions adopt behaviours based on their individual characteristics. For example, some studies have identified various individual behaviours, such as aggressive or defensive driving styles, with different consequences for prevention (Lajunen et al. 2022; Agnoor et al. 2022). However, these studies have not delved into the multicausality of worker behaviour and its impact on occupational accidents.

From a methodological perspective, no research has been found to date that establishes the prevalence of work-related accidents throughout life in worker samples. This research approach could be both interesting and promising, since assessing which workers experience occupational accidents would allow the customization of preventive actions. In addition, this approach would also allow for establishing the possible consequences arising from these accidents. It would not be surprising if a work accident resulted in greater vulnerability to experiencing others in the future.

It is necessary to include the gender perspective in the applied field. The gender gap is worrisome, as there is evidence of large differences in morphology, in access to jobs, and in the distribution of work and family burdens in women (Dilmaghani and Tabvuma 2019). In addition, it is generally accepted that women access positions with higher levels of precarity, less responsibility, and worse contractual conditions due to lack of equity, social and occupational discrimination, and the family burdens to which they are subjected (Undurraga and Hornickel 2020; Van der Lippe et al. 2018).

All of the above is applicable to the service sector, specifically, to the area of cleaning. The absenteeism rate for cleaning services is 7.5% compared to the national average of 5.3%. In addition, the incidence rate (accidents per 100,000 workers) of absenteeism due to work accidents (3480.4) is also above the national average (3408.7) (Ministerio de Trabajo Migraciones y Seguridad Social 2018). A body of evidence reflects that this sector exemplifies the vulnerability of women in the workplace. Cleaning is an activity in which the majority of workers are women and in which men occupy the positions with the greatest responsibilities. Usually, no qualifications are required for its execution, and it is mainly a physical activity. Many of the disabilities, limitations, and absences that are generated are linked to ergonomic aspects derived from the manual handling of loads, forced postures, or the execution of repetitive movements (Larson et al. 2022; Gilchrist and Pokorná 2021). Therefore, the immediate and long-term consequences of work-related accidents can be quite significant.

This study was carried out with women hired to perform cleaning tasks. It first seeks to establish the prevalence of accidents with medical leave throughout the life of these women. The second aim is to identify possible differences (sociodemographic, occupational, psychosocial, perceived health, and personality) associated with women who have experienced an accident at work that involved medical leave in their life. Finally, the third aim is to establish the possible profiles of female workers based on their accident with medical leave history. The purpose of such research is to aid in designing individualized preventive measures that reduce the accident rate.

## Materials and methods

### Subjects

Before starting the investigation, permission was obtained from the occupational safety and health committee and the works council. All participants signed an informed consent form.

The study’s target population comprised 2637 workers, especially cleaning personnel from the service sector, distributed nationwide (Spain). A total of 502 workers participated in the study (19.0%). Of these 502 questionnaires, 217 were obtained in face-to-face training sessions on occupational risk prevention. Among them, a 100% response rate was obtained. The rest (*n* = 285) were collected through distance training submitted via postal mail. A total of 350 distance training mailers were sent out with questionnaires, of which 28 (5.6%) were returned due to postal address error, 13 (2.6%) were incomplete or did not identify the worker, and 24 (4.8%) were not returned by the workers.

The final sample in this study is composed of 455 women working as cleaners. The average age of participants was 49.13 years (SD 9.10). The majority were Spanish (97.4%; *n* = 443) and were single (62.0%; *n* = 225). Most were located in Asturias (43.7%; *n* = 199) or Galicia (20.6%; *n* = 94), and showed no limitations in medical examinations (94.6%; *n* = 210) or disabilities (90.8%; *n* = 413).

### Design

This was a multicentre study with an ex post facto retrospective design (no randomization) of two groups, one of them is a quasi-control group (those women who did not have accidents with medical leave).

### Instruments

The sociodemographic variables used were gender, age, province of residence, nationality, marital status, and recognized disability.

The *SF-36* *Health* *Survey* (SF-36) is a self-reported questionnaire of perceived health that provides a general perspective on individual health status (García et al. 2004; López-García et al. 2003; Vilagut et al. 2005). The 36 questions constitute eight dimensions measured by the questionnaire: (1) bodily pain, (2) mental health, (3) emotional role, (4) social function, (5) physical role, (6) vitality, (7) physical function, and (8) general perception of health status. The scales range between 0 and 100, with 100 being the optimal level of health. This questionnaire is one of the most widely used for evaluating generic health-related quality of life. In Spanish samples, it has proved good construct validity; good predictive validity and sensitivity to change in quality of life (Vilagut et al. 2005). In the present sample, the Cronbach’s alpha coefficient for this questionnaire was 0.807.

The *Mini* *Psychosocial* *Factor* *Method* (MPF) (Ruiz García and Idoate García 2008) performs a simplified psychosocial analysis using a 15-item questionnaire (Montoya-García et al. 2013). The instrument collects information related to (1) work pace, (2) mobbing, (3) human and work relationships, (4) perception of health status, (5) task recognition, (6) autonomy, (7) compensation, and (8) job support from peers and managers. The items are scored using a Likert scale with scores between 1 and 10, which define three levels of risk. Scores lower than 4 imply an obvious risk, between 4 and 7 risk suspicion, and above 7 absence of risk. MPF is a good initial diagnostic method, being quick as well as statistically reliable and valid; in addition, it is ideal for studying populations (Montoya-García et al. 2013) This method can be used in any Spanish work sector (industry, administration, agriculture, tourism, education, etc.) and examples of this with reliable results in improving the workplace include: Consejo Superior de Investigaciones Científicas de España (Higher Council for Scientific Research of Spain), University of Almería, ArcelorMittal, Navarro Health Service, Administrator to train infrastructure (Ruiz García and Idoate García 2008). In the present sample, the Cronbach’s alpha coefficient for this questionnaire was 0.770.

The *Substance* *Use* *Risk* *Profile* (Woicik et al. 2009) assesses, through 23 statements, four dimensions of personality related to risk behaviours: (1) sensitivity to anxiety, (2) hope (vs. depression), (3) impulsivity, and (4) sensation seeking. In a previous study carried out with this specific sample (working women), two additional factors (conducting Exploratory Factor Analysis) were found (Fernández-Suárez 2017). Thus, they have been included in this research (5) sensation of failure and manipulative behaviour and (6) risk behaviours. The higher the score in one dimension is, the greater the presence of the evaluated characteristic. Construct validity, convergent validity, and discriminative validity have been shown to be adequate in samples of adolescents and adults (Krank et al. 2011). In the present sample, the Cronbach’s alpha coefficient for this questionnaire was 0.786.

### Procedure

Once the sample and the assessment instruments were determined, a questionnaire was prepared with the tools described. In addition to the instruments, the instructions for completion, the explanation of the research objectives, and the commitment to confidentiality in relation to the data obtained, along with informed consent, were included. Given the cultural level of the sample, the need to apply simple tests with large font sizes was established, and thus, the use of new technologies was dismissed due to the high rate of digital illiteracy. The questionnaires were analysed by the author and transferred to a data collection form.

The questionnaires were administered at the end of the training in prevention conducted by the company. In the case of face-to-face training, the questionnaire was provided at the end of the course. In the distance learning courses, another envelope was included, stamped with the address of the company, in which the aforementioned training record and the research questionnaire were enclosed.

### Data analysis

The comparison between the different groups (female workers with and without accidents) was performed using the Chi-square test in the case of categorical variables and Student’s *t* test for quantitative variables. In the latter case, the Levene test was performed to verify the homogeneity of the variance (Glass and Stanley 1970).

To calculate the effect size between the two groups, the formula consisting of the quotient between the mean difference of each group and the standard deviation of the total sample was applied (Cohen 1988). In evaluating the magnitude of the effect size, the general recommendations of Cohen (1988) have been applied, which consider the following criteria: *d* = 0.20 (small effect), *d* = 0.50 (moderate effect), and *d* = 0.80 (large effect). It has also been considered that values of approximately 0.30 may be relevant for clinical practice (Borg et al. 1993).

To conduct the multivariate analysis and establish cut-off points between the groups with and without accidents, CHAID (Chi-squared Automatic Interaction Detection) segmentation analysis was used. This technique, developed by various authors, evaluates the discriminant capacity of several independent variables (quantitative or qualitative) on a dependent variable (in this case, the assignment to the accident or non-accident group) through the significance of the *χ*^2^ (Cellard et al. 1967). Therefore, it is a nonparametric test (very robust). For each of the subsamples (nodes), the odds ratio associated with having suffered an accident was calculated. All statistical analyses were performed with the SPSS package (vs. 25.0).

## Results

A total of 23.5% of the workers (*n* = 107) had suffered an occupational accident with medical leave.

### Comparisons between those who had suffered accidents and those who had not

Table 1 compares the sociodemographic variables of the women with and without accidents with leave in her history. Women with accidents were older and in greater proportion married, with medical limitations, with disability and trained in occupational risk prevention than women without accidents.

**Table 1:** Comparison of sociodemographic and work variables among those who have not had and have had accidents with medical leave in the history

	Without accidents (*n* = 348)		With accidents (*n* = 107)		*t* (*df*)	*p*
	Mean	(SD)	Mean	(SD)		
Age	48.51	9.21	51.17	8.48	2.66 (453)	0.008
	*n*	%	*n*	%	*χ*^2^ (*df*)	*p*
Marital status	273		90			
Single	185	67.8%	40	44.4%	16.4 (2)	0.000
Married/long-term relationships	77	28.2%	46	51.1%		
Separated	11	4.0%	4	4.4%		
Limitations of medical examination	152		70			
Without limitations	148	97.4%	62	88.6%	7.3 (1)	0.007
With limitations	4	2.6%	8	11.4%		
Disability	348		107			
Without disability	325	93.4%	88	82.2%	12.1 (1)	0.000
With disability	23	6.6%	19	17.8%		
Training in occupational risk prevention	348		107			
No	94	27.0%	7	6.5%	19.9 (1)	0.000
Yes	254	73.0%	100	93.5%		

Table 2 shows the comparisons in perceived health, psychosocial factors, and personality variables among those who have had or have not had an accident with medical leave. In all dimensions of the perceived health (*SF-36*), women who suffered medical leave accidents presented lower scores (worse perception) compared to women who did not suffer medical leave accidents. In the case of psychosocial risks (MPF), women who experienced medical leave accidents presented lower scores (higher psychosocial risk) across 5 dimensions and higher score (lower risk) in Mobbing than those who have not experienced any accidents. Women with medical leave accidents presented higher scores in sensitivity and anxiety. These differences were statistically significant.

**Table 2:** Comparison of perception of health status, psychosocial factors, and personality variables among those who have had or have not had accidents with medical leave

	Without accidents (*n* = 348)		With accidents (*n* = 107)		*t*	(*df*)	*p*	*d*
	Mean	(SD)	Mean	(SD)				
SF-36 health survey								
Physical function	92.87	13.34	79.99	24.15	5.27	126.7	0.000	0.74
Physical role	91.64	24.98	67.86	43.81	5.31	125.2	0.000	0.74
Pain in the body	81.56	21.18	62.86	31.47	5.71	134.3	0.000	0.74
Social function	92.15	15.66	80.31	25.70	4.50	129.8	0.000	0.62
Vitality	71.59	19.28	59.98	23.19	4.68	152.6	0.000	0.56
Mental health	81.65	16.50	74.00	21.77	3.33	144.4	0.001	0.42
Emotional role	93.80	21.45	85.72	31.64	2.45	134.4	0.016	0.33
Mini psychosocial factors	(*n* = 342)		(*n* = 99)					
Rhythm of work	6.00	1.74	5.22	1.92	3.84	439	0.000	0.41
Perception of health status	6.42	1.97	5.56	2.34	3.37	140.4	0.001	0.37
Autonomy	6.22	1.68	5.83	1.66	2.05	439	0.041	0.23
Support	5.89	0.99	5.67	0.97	1.99	439	0.047	0.22
Recognition of the task	5.23	2.45	4.67	2.51	1.99	439	0.048	0.22
Mobbing	5.26	0.97	5.50	1.07	2.03	148.1	0.044	0.22
Compensation	6.08	1.89	5.71	1.96	1.74	439	0.083	0.19
Relations	5.95	1.52	6.00	1.38	0.31	439	0.760	0.04
Substance-use risk profile	(*n* = 348)		(*n* = 107)					
Sensitivity to anxiety	10.26	4.25	11.55	4.82	2.7	453	0.008	0.29
Hope	20.64	4.99	19.70	5.93	1.5	154.9	0.141	0.18
Searching for sensations	6.28	2.29	5.99	2.21	1.1	453	0.251	0.13
Impulsivity	6.90	2.49	7.35	2.85	1.6	453	0.115	0.17
Failure manipulation	5.92	0.72	5.82	0.94	1.0	144.1	0.314	0.13
Risky behaviours	4.93	2.28	4.52	2.42	1.6	453	0.112	0.18

From a qualitative perspective, women who have suffered accidents with medical leave present a greater proportion of an evident risk in three of the evaluated psychosocial factors: task recognition, perception of health status, and work rate (Table 3).

**Table 3:** Comparison of psychosocial factors among those who have not had and have had accidents with medical leave in the history based on psychosocial risk (MPF)

	Without accidents (*n* = 342)		With accidents (*n* = 99)		*χ*^*2*^	(*df*)	*p*
	*n*	%	*n*	%			
Recognition of the task							
Obvious risk	76	22.2	35	35.4			
Suspicion of risk	217	63.5	51	51.5	7.1	(2)	0.028
Absence of risk	49	14.3	13	13.1			
Perception of health status							
Obvious risk	23	6.7	22	22.2			
Suspicion of risk	228	66.7	55	55.6	20.1	(2)	< 0.001
Absence of risk	91	26.6	22	22.2			
Rhythm of work							
Obvious risk	20	5.8	20	20.2			
Suspicion of risk	250	73.1	68	68.7	21.7	(2)	< 0.001
Absence of risk	72	21.1	11	11.1			
Compensation							
Obvious risk	36	10.5	13	13.1			
Suspicion of risk	224	65.5	64	64.6	0.6	(2)	0.749
Absence of risk	82	24.0	22	22.2			
Support							
Obvious risk	9	2.6	6	6.1			
Suspicion of risk	308	90.1	90	90.9	4.9	(3)	0.087
Absence of risk	25	7.3	3	3.0			
Autonomy							
Obvious risk	11	3.2	5	5.1			
Suspicion of risk	251	73.4	79	79.8	3.5	(2)	0.169
Absence of risk	80	23.4	15	15.2			
Relationships							
Obvious risk	21	6.1	5	5.1			
Suspicion of risk	261	76.3	80	80.8	0.9	(2)	0.643
Absence of risk	60	17.5	14	14.1			
Mobbing							
Obvious risk	10	2.9	0	–			
Suspicion of risk	317	92.7	91	91.9	4.9	(2)	0.086
Absence of risk	15	4.4	8	8.1			

*MPF* Mini Psychosocial Factor Method

### Results of the CHAID analysis

**Fig. 1:**
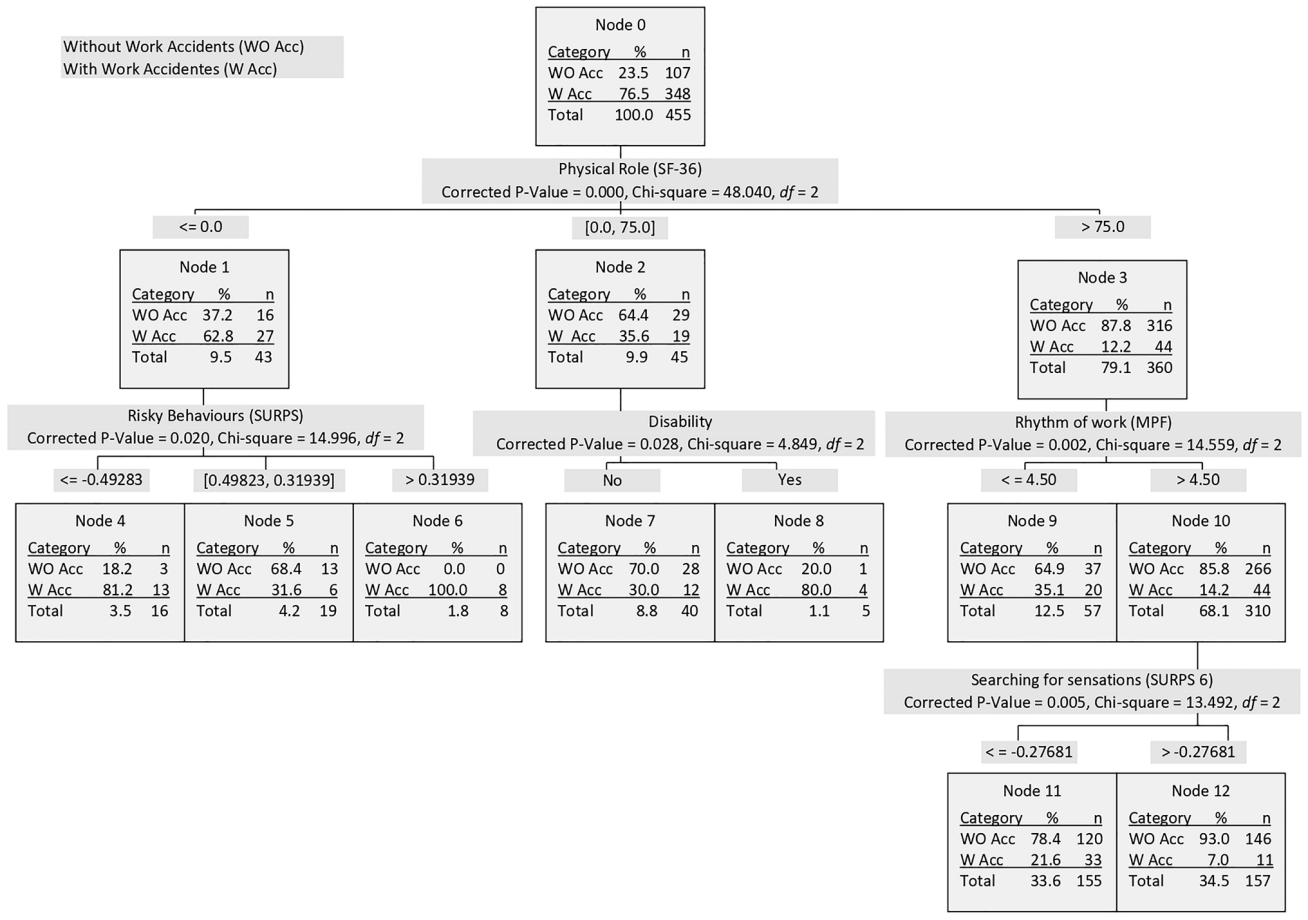
Results of the CHAID analysis

From a multivariate perspective, the 455 workers were grouped into 12 subsamples, among which in all cases, there were statistically significant differences between those who had suffered accidents with medical leave and those who had not. At the first level, the physical role classifies all cases into three categories (nodes 1, 2, and 3; Fig. 1). Therefore, 62.8% of women who scored 0 in Physical Role (SF-36), 35.6% of women who scored up to 75, and 12.2% who scored higher than 75% had suffered work accidents with medical leave. Each of these nodes, in turn, is divided into a second level as a function of the risky behaviours (for node 1, nodes 4–6 with the highest rate of accident in women who scored higher than 0.3193 corresponding to node 6), the existence of a disability (for node 2, nodes 7 and 8), and the differences in the pace of work (for node 3, nodes 9 and 10). From node 10, and again as a function of the scores in the search for sensations, two new nodes can be differentiated (node 11 with 21.6% with women who had suffered accident with medical leave and node 12 regarding with 7.0%).

Not all nodes represent the same proportion of women. Therefore, Table 4 shows the percentage of each node with respect to the total number of women and the injured. In addition, the odds ratio associated with the probability of having suffered an accident with medical leave for each node is included. In node 6, all women had suffered an accident with a probability of 15.9 times; in node 8, this probability was 13.5. At the other extreme, in nodes 3, 10, and 12, the associated probability is almost nonexistent (0.2).

**Table 4:** Percentage of total people, accidents, and odds ratios associated with each node (accidents with medical leave in the history)

	People over total (*N* = 455)		Accidents over total (*N* = 107)		Odds ratio
	*N*	%	*N*	%	
First level					
Node 1	43	9.5	43	25.2	7.0
Node 2	45	9.9	16	15.0	1.9
Node 3	367	80.7	64	59.8	0.2
Second level					
Node 1	43	9.5	43	25.2	7.0
Node 4	16	3.5	13	12.1	15.9
Node 5	19	4.2	6	5.6	1.5
Node 6	8	1.8	8	7.5	∞
Node 2	45	9.9	16	15.0	1.9
Node 7	40	8.8	12	11.2	1.4
Node 8	5	1.1	4	3.7	13.5
Node 3	367	80.7	64	59.8	0.2
Node 9	57	12.5	20	18.7	1.9
Node 10	310	68.1	44	41.1	0.2
Third level					
Node 10	310	68.1	44	41.1	0.2
Node 11	153	33.6	33	30.8	0.8
Node 12	157	34.5	11	10.3	0.2

## Discussion

In this study, which assessed the prevalence of work accidents with sick leave in Spain, it was found that one in four women in the cleaning sector had suffered at least one accident in their working life. From the point of view of safety and health at work, this tendency is a concerning fact that has not been documented so far.

In addition, we have identified variables that differentiate between those who have had an accident and those who have not and have described profiles of women with a greater propensity to work-related accidents. Regarding the variables analysed individually, beginning with the sociodemographic variables, it was found that female workers who had experienced accidents with medical leave in their history were 2.66 years older than those who have not suffered accidents. This figure is in line with the study by Payne and Doyal (2010), which analyses the ageing of working women and its influence on occupational accidents, as well as other studies that relate ageing with absenteeism and production (Botti et al. 2022; Andersen et al. 2021; Sundstrup et al. 2020; Stoesz et al. 2020). A larger proportion of injured workers is married or in a long-term relationship. This result supports the results of previous studies which indicate that the married population usually has a lower risk of suffering serious accidents but a higher incidence of minor accidents (Afshar et al. 2022; Farahbod et al. 2021; Saranjam et al. 2020). This may be related to other variables such as impulsivity (ability to maintain stable relationships) or age (the longer the relationship, the older).

With respect to the analysed work variables, women in accidents presented with a greater proportion of medical limitations, disability, and training. Medical limitations are the result of problems in health status; therefore, if no adaptations are made, the chances of suffering damage could be increased. Regarding disability, to date, studies have focused on disability as a consequence of occupational accidents (Ho and Lin 2022; Stemn and Krampah 2022; Sun et al. 2022). However, this condition should be included as a possible cause, especially in samples such as the one evaluated, with mild accidents of musculoskeletal origin. Finally, in relation to training, the organization reinforces the training of injured workers as a preventive measure, so training can also be a consequence of the accident.

In relation to perceived health status (Bodner et al. 2014; Zhang et al. 2015), a statistically significant relationship was found between the perception of health status and accidents. The injured women had a worse situation in all areas evaluated (with very high effect sizes), including physical function, physical role, body pain, social function, vitality, mental health, and emotional role. The subjective perspective in health analysis and the evaluation of psychosocial risks is not currently included in Spanish policies regarding work security and health. The inclusion of this novel perspective will allow recommendations of an organizational and/or psychosocial nature to be made, such as the need to increase supervision or strengthen the training of certain workers (López-Goñi et al. 2022). However, caution should be exercised in assessing the result, since, again, health may be the cause or the consequence of the accident. This relationship requires further investigation.

Regarding psychosocial factors, important differences have also been found. The greatest effect size was found in the work rhythm (effort during the task and its intensity) (Briguglio et al. 2021). The relationship between work rhythm and the pathologies of the sample, especially musculoskeletal, is consistent. It is an eminently physical activity; therefore, a High Work Pace will increase the possibility of suffering an occupational accident with consequences on the musculoskeletal system. The higher the rate of movement, the greater the probability of injury. In addition, the greater work rate, the higher probability of suffering accidents (Bao et al. 2016). The other psychosocial factors also present important differences, and it would be desirable to continue the investigation.

Among the dimensions of personality evaluated, the sensitivity to anxiety was higher in injured women. Anxiety, its relationship to pathologies derived from work environments, and its influence on human behaviour have been the subject of substantial investigation. For example, high levels of anxiety in novice drivers have been found to lead to increased errors and, consequently, a higher number of accidents (Chalmers et al. 2021; Sun et al. 2022). Finally, from a multivariate perspective, specific subsamples have been found to have a high probability of having suffered accidents.

In any case, caution should be exercised in generalizing the results obtained. The sample in this study has not been randomly selected. All possible cases of workers with accidents in the past have been included to develop differential profiles and obtain a sample with a high number of occupational accidents. Therefore, the accident rate is higher than in the reference population. Furthermore, this research has focused on women who work in cleaning activities. In future studies, samples should be used from other work environments and should include men. Additionally, new variables could be included, such as common contingencies. In addition, the design of the study was retrospective, so causality cannot be established in terms of the relationships found. However, the study allows for designing specific and individualized preventive actions for female workers with a greater tendency to experience work accidents.

From a preventive perspective, due to the high probability of having suffered accidents of this population, a special attention should be paid to workers with low physical roles and high scores in risk behaviours. All women in this node had accidents. Another subsample at great risk is that of female workers with low physical role and low score in risky behaviours. The understanding of the relationship between the influences of the two extreme values of the risk behaviour dimension is an interesting line of future research. Women with a medium physical role and disability are also an at-risk population.

## Conclusion

In general, it was found that the individual characteristics of female workers in the different categories are associated with different accident rates. However, preventive measures and actions in occupational safety and health are usually homogeneous, generic, and not adapted at the individual level. If people are heterogeneous, with different characteristics, we should design flexible occupational risk prevention systems, with individualized interventions, in which certain profiles will demand greater efforts than others.

## Data Availability

The data are not publicly available due to them containing information that could compromise research participant privacy/consent.
